# Climate change and mental health of Indigenous peoples living in their territory: a concept mapping study

**DOI:** 10.3389/fpsyt.2023.1237740

**Published:** 2023-11-06

**Authors:** Antonio Jose Grande, Ieda M. A. V. Dias, Paulo T. C. Jardim, Alessandra Aparecida Vieira Machado, Jacks Soratto, Maria Inês da Rosa, Leonardo Roever, Luciane Bisognin Ceretta, Xanthi Zourntos, Seeromanie Harding

**Affiliations:** ^1^Medicine School, State University of Mato Grosso do Sul, Mato Grosso do Sul, Campo Grande, Brazil; ^2^Public Health Department, Federal University of Rio Grande do Sul, Porto Alegre, Rio Grande do Sul, Brazil; ^3^Public Health Department, Universidade do Extremo Sul Catarinense, Santa Catarina, Criciúma, Brazil; ^4^Department of Clinical Research, Brazilian Evidence-Based Health Network, Uberlândia, Brazil; ^5^Gilbert and Rose-Marie Chagoury School of Medicine, Lebanese American University, Beirut, Lebanon; ^6^Department of Population Health Sciences, School of Population Health and Environmental Sciences, Faculty of Life Sciences and Medicine, King's College London, London, United Kingdom

**Keywords:** Indigenous, climate change, mental health, concept mapping, community based participatory research

## Abstract

**Background:**

The alarming increase in annual deforestation rates has had devastating consequences in climate change, and it is affecting Indigenous people, who depend entirely on the land for survival and has also weakened the rainforest's crucial role in stabilizing the global climate. Recognizing and respecting Indigenous people's needs and social, economic, and historical conditions influence health and healthcare. This study aimed to conduct online concept mapping workshops with university students to identify perceived important and feasible actions for improving the mental health of Indigenous people living in their territory in association with climate change.

**Methods:**

Concept mapping, a participatory mixed methodology, was conducted virtually with 20 Indigenous students at two universities in Brazil. A focus prompt was developed from consultations with Indigenous stakeholders and read—“To improve the mental health of Indigenous peoples in their territory during climate change crises, it is necessary to….”

**Results:**

University students organized 42 unique statements in 6 clusters that cover a wide range of topics: family support, 0.68 (SD 0.19); respect and understanding, 0.37 (SD 0.08); improvement actions, 0.52 (SD 0.07); public policies in favor of Indigenous people's mental health, 0.24 (0.09); health actions, 0.15 (SD 0.08); Indigenous training in health and its importance in improving mental health 0.32 (SD 0.07).

**Conclusion:**

These clusters range from community initiatives, public policies, health actions, and strengthening professional services in Indigenous communities. These all provide numerous concrete ideas for developing interventions designed to address mental health challenges associated with climate change.

## Introduction

Climate change has damaging consequences on global and local scales and poses significant threats to health, natural resources, and vulnerable populations, particularly Indigenous populations. The Latin America and the Caribbean (LAC) region is home to over 40 million Indigenous people (1). Brazil, one of the four countries with the largest Indigenous populations, has ~1 million Indigenous people spread among 300 tribes, of which ~100 are uncontacted (2).

Globally, Indigenous peoples experience adverse health outcomes compared with their non-Indigenous counterparts. For example, Anderson et al. (3) report a gap in life expectancy at birth (that is lower in Indigenous than the non-Indigenous population) of more than 5 years. Infant mortality rates for Indigenous infants are more than twice of those observed for non-Indigenous populations in Brazil, Colombia, Peru, and Venezuela (4). Disparities were also noted for stunting among the <5-year-olds and low birth weight in some countries; poverty, poor education levels, employment status, and access to health services contributed to the health disparities (5). Suicide rates in Indigenous populations vary globally, and the disparities between Indigenous and non-Indigenous populations are substantial (6). Discrimination, poverty, ostracization from the mainstream, and identity crises are key factors that influence this widening gap (7, 8).

As stated in the Lancet Commission on Adolescent Health, Indigenous young people face even more significant challenges to health and wellbeing, adversely impacting their abilities to cope with the stressors of life (3). Youth suicide is the second leading cause of mortality among 15–29-year-olds and disproportionately affects Indigenous youth (6). Indigenous children (5–17 years old) in Australia die from suicide at a rate of five times more than their non-Indigenous peers (9), and in New Zealand, the suicide rate in Maori youth aged 15–24 years is more than twice as much as in their non-Maori peers (10). Most interpretations of this gap highlight the persistent social and economic disadvantage experienced by Indigenous youth (10). The epidemic of youth suicide is relatively recent in some Indigenous cultures, with men accounting for most suicides and those aged 15–24 years having the highest suicide rates than any other age group (11). Risk factors include mental health disorders, stressful life events, substance abuse, and poor physical health, all occurring at disproportionately higher rates in Indigenous populations (12).

Adolescents and young people have long been excluded from participating in political and other policy processes, corroborating their feelings of powerlessness and potentially contributing to their high burden of poor mental health (13, 14). The threat of irreversible and long-term climate change is an additional mental health risk factor for youth, particularly those already vulnerable to non-environmental effects (15). Findings from the *Xunati Uti* study in Mato Grosso do Sul, Brazil showed that Indigenous university students perceived their health and happiness to be influenced by family life, friendships, the state of nature, and belonging to a strong community (16). Developing processes that facilitate the involvement of Indigenous youths to advocate for their environmental security may positively impact their health and wellbeing (15, 17).

Indigenous people's rights have been increasingly recognized through international organizations such as the United Nations Permanent Forum on Indigenous Issues, which has a permanent forum for youth (13, 18). In 2017, the World Health Organization (WHO) published “*Policy on Ethnicity and Health*”, highlighting the need for institutional and community capacity to produce sufficient quality data and generate evidence for policy-making concerning health inequities experienced by Indigenous peoples (13, 18). The Pan American Health Organization (PAHO's) “*Health Plan for Indigenous Youth*” identifies several priority areas, including access to intercultural health services, traditional medicines, mental health, disabilities, and violence. Despite these promising policy actions, health and wellbeing disparities between Indigenous and non-Indigenous people persist (19, 20).

The present study uses a participatory mixed-methods concept mapping (CM) approach to identify important and feasible factors to improve Indigenous people's mental health associated with climate change.

## Methods

### Ethics

Ethical approval for this study was obtained from the National Research Ethics Commission (CONEP), Protocol CAAE 36372820.0.0000.8027 from May 25, 2021. All participants signed an agreement form.

### Recruitment

One researcher at Universidade Estadual de Mato Grosso do Sul (UEMS) and one at Universidade Federal de Rio Grande do Sul (UFRGS) advertised the study among Indigenous students. Concept mapping activities took place between August and September 2021.

This was a convenience sample; students attended the authors' university (AJG and IMAVD); four Indigenous youths are offered a scholarship each year to undertake each undergraduate course (e.g., Medicine, Nursing, and Psychology). A total of 20 Indigenous university students were invited to participate in the study. They were identified across six different ethnicities in South, Middle-West, and Northeast Brazil.

### Concept mapping

Four meetings were conducted virtually through Google Meet, each lasting 30 min to 2 h. Participatory CM is a structured process that generates statements from participants' discourse, which are later sorted and rated for importance and feasibility (21). The key steps included the following: (1) brainstorming to a focus prompt, (2) sorting and rating, and (3) a map interpretation session. Data collection occurred during the COVID-19 pandemic, from August 2021 through September 2021. All meetings were facilitated by researchers.

**Figure 1:**
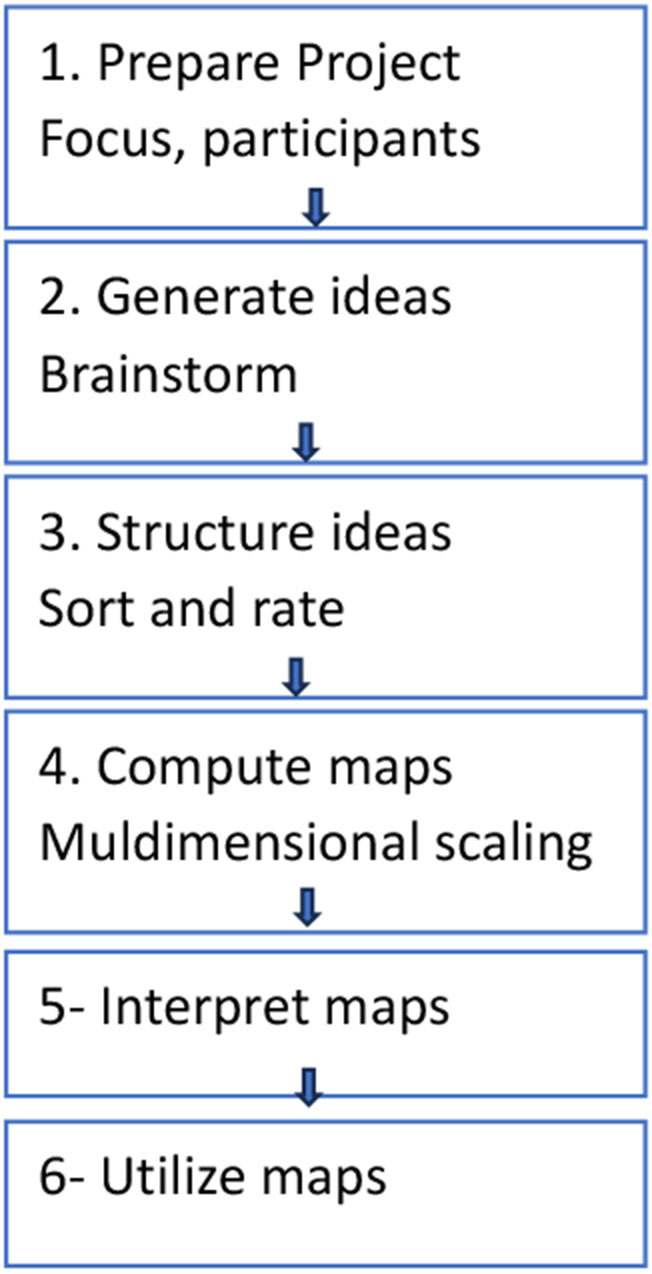
Concept mapping research steps.

Figure 1 presents each step of the concept mapping.

### Brainstorming

The first meeting was held to explain the objective of the research, to gather participant consent, and to conduct the brainstorming session. During the first meeting, we asked participants to complete an online form of demographic questions. Once participants completed the demographic portion, the brainstorming began by sharing the focus promptly.

A 1-h brainstorming meeting was individually conducted with 20 Indigenous university students. The brainstorming activity was guided by the focus prompt:

“*To improve the mental health of Indigenous peoples in their territory during climate changes crisis, it is necessary to*...”

Participants took note of their statements during the brainstorming session and sent statements via email to the leading researcher after the meeting. In total, 86 participant statements were generated and then narrowed down by the research team. Researchers then removed repeated statements and, in discussion with the participants, amended some for clarity. The final list included 42 statements.

### Sorting and rating

All participants who took part in the brainstorming activity were involved in sorting and rating. Each participant was invited to individually organize the 42 statements into clusters and label the clusters appropriately on an online Google Form. Participants were then asked to rate each statement on the Google Form according to their perception of importance and feasibility, using a 5-point Likert scale. The questions read, “*How important are each of the following statements regarding climate change and the adaptation of Indigenous people?*” and “*How feasible is it to implement each of the following statements into practice?*” The 5-point rating scales were 1 = Relatively unimportant, 2 = Somewhat important, 3 = Moderately important, 4 = Very important, 5 = Extremely important, and for the feasibility of achieving a positive change 1 = Not at all feasible, 2 = Somewhat feasible, 3 = Moderately feasible, 4 = Very feasible, 5 = Extremely feasible.

The data collected from the completed forms were inputted into the Concept Systems Global Max software to conduct three-step multivariate statistical analyses of participant data, represented in two multidimensional maps.

A third meeting was held to present and discuss the multidimensional maps with participants and agree on appropriate cluster maps and labels.

### Feedback session with participants

A final meeting was held to present the results to participants, to encourage participant validation of the analyzed data, and give their feedback on the results. The participants were encouraged to make any changes they felt necessary that would improve the clarity of the representation of the results. The participants analyzed the six-cluster map and agreed it best represented the key ideas, themes, and their unique connectivity.

### Data analysis

Quantitative multivariate analyses of clusters and ratings were conducted to produce multiple visual maps of perceived actionable priorities. Analyses were performed using the Group Wisdom TM software (22). Two researchers manually entered all the data collected through emails and Google Forms, double-checked the Concept Systems Global Max software, and analyzed the data in three steps.

A matrix of similarities was generated to check the statements and the labels given to each statement group. Multidimensional scaling (MDS) analysis created a two-dimensional “point map”. Each statement was represented as a numbered point, with points closest together more conceptually similar. The stress value of the point map is a measure of how well the MDS solution maps the original data, indicating a good fit. Stress values range from 0 to 1, with lower values indicating better fit. The acceptable range for stress values is 0.205–0.365 (23). Hierarchical cluster analysis was then used to delineate clusters of statements (points) that are conceptually similar and create associated cluster maps using the grouping of statements from the point map. Following feedback sessions with participants, a 6-cluster map was agreed to represent the 42 statements appropriately. The Indigenous participants determined cluster labels. Clusters with low bridging values indicate high agreement among participants in the clustering of statements. Go-Zone graphs showed the most actionable (high importance and high feasibility) and least actionable (low importance and low feasibility) statements.

The research team performed cluster analysis and multidimensional scaling to allow for an illustration of the sorted data across all participants in a spatially oriented map of the statements or concept map (24).

## Results

### Participant characteristics

In this study, 20 Indigenous university students from Mato Grosso do Sul and Rio Grande do Sul States participated in all steps of this study. In total, 60% of participants were female individuals (*n* = 12) and 90% were single (*n* = 18). Half of the stakeholders hail from Kaingang ethnicity (*n* = 10) and receive a university stipend (*n* = 13). In total, 60% considered themselves rural Indigenous individuals (*n* = 12) (living in a rural area).

Participant sociodemographic characteristics are presented in Table 1.

**Table 1:** Demographic characteristics of the stakeholders.

**Age**	**Mean**	**Standard deviation**	
	25.25	4.63	
		**N**	**%**
Sex	Male	8	40
	Female	12	60
Marital status	Single	18	90
	Married	2	10
Ethnicity	Kaingang	10	50
	Pitaguary	1	5
	Terena	3	15
	Atikum	4	20
	Arapium	1	5
	Tabajara	1	5
Income type	Scholarship	13	65
	Parents help	3	15
	None	4	20
Indigenous	Urban	8	40
	Rural	12	60

### Cluster analysis

**Figure 2:**
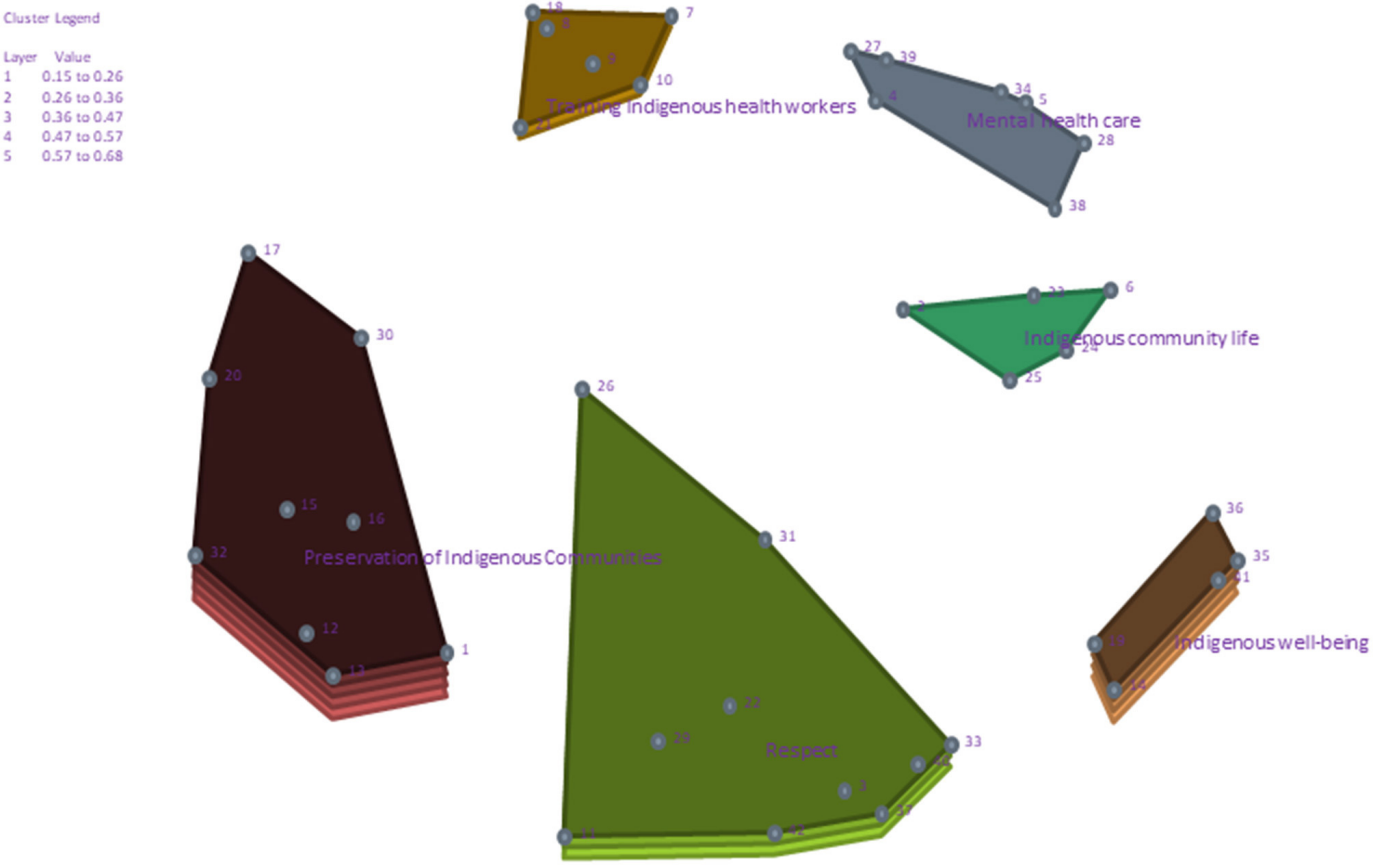
Cluster concept map showcasing the factors that stakeholders felt were important and feasible to influence the protection of their mental health in response to climate change.

**Figure 3:**
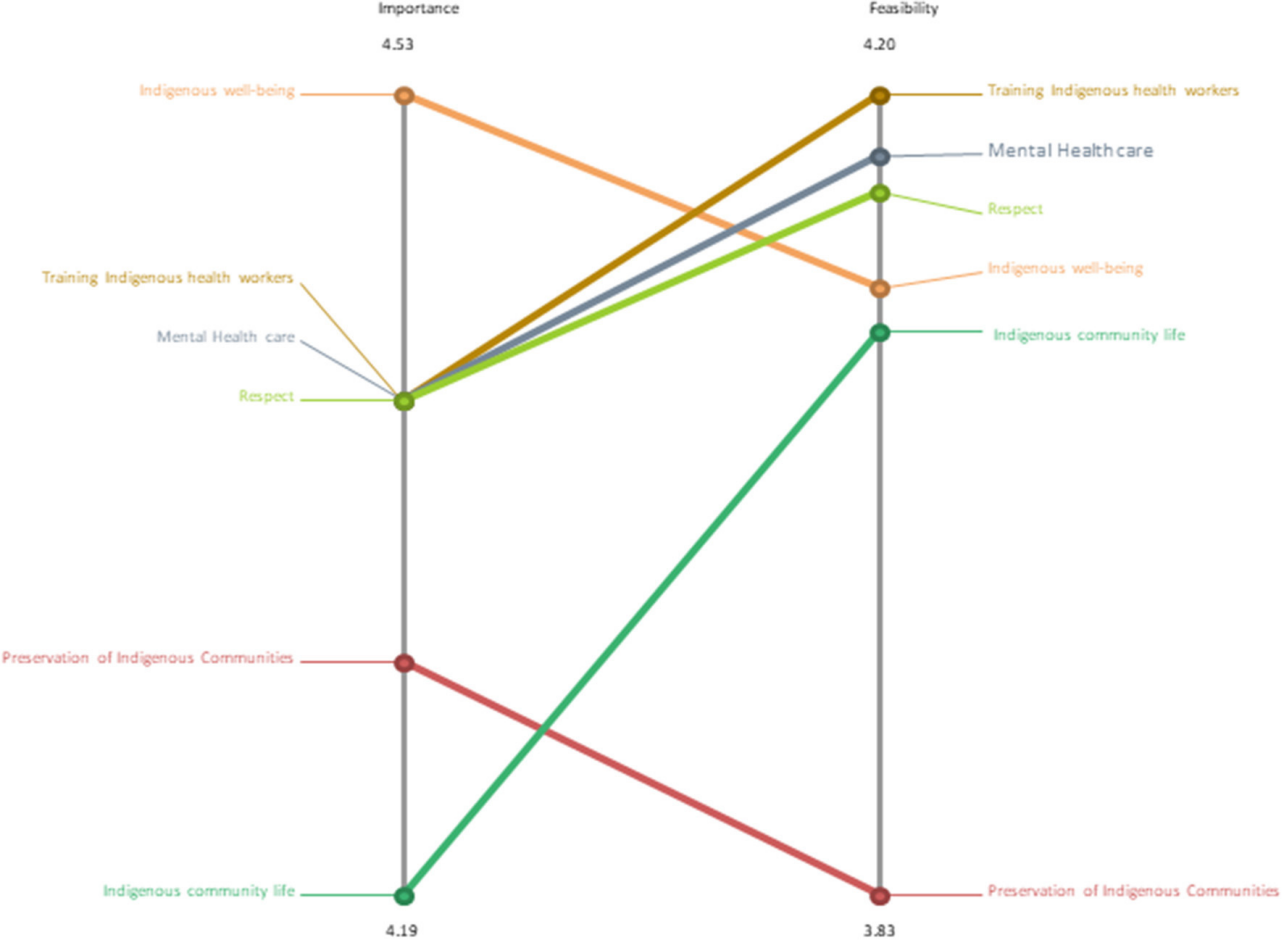
Pattern match of average perceived importance and feasibility ratings.

Representative statements and bridging values for each statement and cluster are presented in Table 2. Stakeholders decided to organize the statements in a final 6-cluster map with a stress value of 0.32: community and cultural support, 0.68 (SD 0.19); building respect and understanding, 0.37 (SD 0.08); fostering change, 0.52 (SD 0.07); public policy action, 0.24 (0.09); health actions, 0.15 (SD 0.08); strengthening professional skills, 0.32 (SD 0.07). Figure 2 shows the visual representation of the clusters as factors that students felt influenced the protection of their mental health in the context of climate change. The layers of each cluster reflect the degree of agreement among stakeholders, with fewer layers representing a higher agreement across stakeholders. Figure 3 illustrates the perceived importance and feasibility of the clusters. The highest agreement across stakeholders in the cluster of statements was health actions (0.15; SD: 0.08), public policy actions (0.24; SD: 0.09), and strengthening professional skills (0.32; SD: 0.07).

**Table 2:** Representative statements for each cluster.

**Cluster statements**	**Bridging value [mean (SD)]**	***N***	**Importance [mean (SD)]**	**Feasibility [mean (SD)]**
**Community and cultural support**	0.68 (0.19)	9	4.53 (0.64)	3.83 (1.16)
1. Preserve the territory for a good quality of life and wellbeing	0.47		4.55 (0.67)	4.15 (0.93)
12. Demarcate our territories to preserve mental health	0.63		4.50 (0.67)	3.85 (1.27)
13. Promote the security of having your land demarcated	0.66		4.35 (0.73)	3.85 (1.27)
15. Prevent climate change from affecting our eating habits	0.54		4.25 (0.83)	3.35 (1.27)
16. Prevent climate change from affecting the livelihoods of families in the village	0.45		4.15 (0.85)	3.50 (1.32)
17. Prevent Indigenous people from leaving to look for work outside the village	0.99		3.55 (0.97)	3.40 (1.47)
20. Promote ways of transmitting knowledge from the oldest to the youngest	1		4.40 (0.73)	4.2 (0.83)
30. Working with non-Indigenous society to reduce prejudice against Indigenous peoples	0.76		4.40 (0.73)	4.00 (1.12)
32. Preserving or restoring the environment so that there is a prospect of continuing the existence of Indigenous communities	0.59		4.45 (0.67)	4.15 (0.99)
**Building respect and understanding**	0.37 (0.08)	10	4.40 (0.72)	4.16 (1.15)
3. Respect the way of life of Indigenous peoples	0.29		4.65 (0.48)	4.40 (0.99)
11. Understand that our territory represents our spirit, so everything that violates our territory affects us	0.49		4.40 (0.58)	4.40 (0.94)
22. Show the importance of our people and culture to society	0.38		4.35 (0.73)	4.35 (0.93)
26. Generate own income	0.41		4.40 (0.73)	3.90 (1.21)
29. Increasing the protection of Indigenous peoples and not making the laws that protect them more flexible	0.42		4.35 (0.85)	4.00 (1.21)
31. Promoting safety when seeking study in cities	0.32		4.30 (0.78)	3.65 (1.27)
33. Promote belonging to their respective people	0.47		4.10 (0.83)	3.90 (0.79)
37. Respect the specificities and individualities of Indigenous peoples	0.36		4.40 (0.73)	4.25 (1.02)
40. Recognizing the Indigenous as a citizen	0.24		4.50 (0.81)	4.20 (1.01)
42. Respect the Indigenous history, tradition, religion, and customs	0.34		4.55 (0.67)	4.50 (1.00)
**Fostering change**	0.52 (0.07)	5	4.53 (0.64)	4.13 (1.15)
14. Ensuring the safety of Indigenous people who suffer violence and prejudice	0.47		4.70 (0.46)	4.05 (1.32)
19. Ensure work, land, and respect	0.46		4.40 (0.80)	3.80 (1.28)
35. Ensuring access to basics in relation to public health	0.52		4.60 (0.49)	4.30 (1.03)
36. Create public policies to better serve Indigenous peoples	0.65		4.55 (0.59)	4.40 (0.82)
41. Ensuring access to jobs, fair wages, hygiene, food, and health conditions	0.5		4.40 (0.86)	4.00 (1.30)
**Public policy action**	0.24 (0.09)	5	4.19 (0.75)	4.09 (1.00)
2. Have a work focused on the good life within the Indigenous community	0.13		4.25 (0.77)	4.15 (1.04)
6. Develop projects for insertion and understanding of Indigenous culture with knowledge exchanges	0.34		4.20 (0.60)	3.95 (0.83)
23. Create activities that occupy the Indigenous people who stay in the village	0.14		4.20 (0.81)	3.95 (1.23)
24. Create community garden	0.28		4.10 (0.77)	4.25 (0.91)
25. Planting fruit trees	0.31		4.20 (0.81)	4.15 (0.99)
**Health actions**	0.15 (0.08)	7	4.40 (0.67)	4.13 (0.98)
4. Create workshops and lectures focussed on the health of the body and mind	0		4.30 (0.71)	4.35 (1.04)
5. Create mental healthcare programs according to each Indigenous culture	0.14		4.45 (0.67)	4.10 (0.91)
27. Promote actions involving people with alcohol problems	0.12		4.50 (0.67)	4.03 (0.98)
28. Conduct work with Indigenous youth to understand their anxieties and fear	0.23		4.35 (0.65)	4.05 (0.83)
34. Conducting rounds of conversations and conferences in the villages through a psychologist, a social worker, and an environmental entity	0.16		4.25 (0.70)	4.15 (1.04)
38. Promote social actions by professionals in all Indigenous territories	0.28		4.35 (0.73)	4.00 (1.03)
39. Identifying people in psychologically vulnerable situations	0.15		4.60 (0.58)	4.25 (1.02)
**Strengthening professional skills**	0.32 (0.07)	6	4.40 (0.71)	4.20 (0.94)
7. Breaking the taboo on mental illness that exists within Indigenous territories	0.26		4.20 (0.81)	4.10 (1.12)
8. Encourage the training of Indigenous people in health areas who work with psychological illnesses	0.31		4.45 (0.67)	4.20 (0.89)
9. Improve communication between professionals and patients	0.31		4.40 (0.80)	4.50 (0.76)
10. Improve treatment adherence	0.26		4.40 (0.66)	4.15 (0.81)
18. Understand that physical and mental health are intertwined	0.33		4.50 (0.67)	3.95 (1.05)
21. Welcoming and sharing knowledge between professionals and the Indigenous population	0.46		4.45 (0.67)	4.30 (1.03)

- Community and cultural support means that access and policies should be offered to Indigenous to ensure work and respect, for example, the government provides opportunities through economic and social policies for jobs that respect the Indigenous way of life. - Building respect and understanding means their own way of living and working with respect to their culture, for example, they have their own time to do things, unlike non-Indigenous people who carry out several activities at one time. - Fostering changes means action that should be done to improve basic life conditions for Indigenous people, for example, government policies that guarantee access to food and basic sanitation. - Public policy action means a border concept of living in the community for health promotion, for example, encouraging the planting of fruit trees and valuing the use of plants as therapeutics. - Health action means education in health and diseases, for example, understanding the mental health problems happening with the Indigenous population. - Strengthening professional skills means improvement in the communication of the professionals with Indigenous people, for example, improving the communication process in relations between Indigenous and non-Indigenous people.

Each of the cluster's statements was assessed and graded for importance and feasibility on a 5-point scale: (1) not feasible at all; (2) not very feasible; (3) neither feasible nor not feasible; (4) feasible; and (5) very feasible. Figure 3 presents the pattern match of average perceived importance and feasibility ratings.

### Community and cultural support; building respect and understanding; fostering change

The cluster **community and cultural support** had a BV of 0.68 (SD 0.19) and nine statements with a mean rating for importance of 4.29 (SD 0.76) and feasibility of 3.83 (SD 1.16). The statements reflect the need for teamed-up support from local and national systems to improve mental health. The cluster **building respect and understanding** had a BV of 0.37 (SD 0.08) and 10 statements with a mean rating for importance of 4.40 (SD 0.72) and feasibility of 4.16 (SD 1.15). The statements reflected the importance of building the capacities of community capital, such as financial, cultural, and environmental capital. The cluster **fostering change** had a BV of 0.52 (SD 0.07) with five statements with mean ratings for importance of 4.53 (SD 0.64) and feasibility of 4.13 (SD 1.15). The students' statements emphasized the need for government support in safeguarding their communities, whether through policies or equitable access to basic sanitation services.

### Public policy action, health actions, and strengthening professional skills

The cluster **public policy action** had a BV of 0.24 (SD 0.09); the five statements have mean ratings for importance of 4.19 (SD 0.75) and feasibility 4.09 (SD 1.00). This cluster highlighted the students' intent to be involved in co-developing mental health interventions centered on Indigenous traditional knowledge, such as establishing community gardens. This approach honors the reciprocal relationship between humans and the earth. The cluster **health actions** had a BV of 0.15 (SD 0.08) and mean ratings for importance of 4.40 (SD 0.67) and feasibility of 4.13 (SD 0.98). The seven statements are conceptually similar to the above cluster and reemphasise the urgency for multisectoral collaboration to ensure mental health policies' successful implementation and sustainability. The cluster **strengthening professional skills** had a BV of 0.32 (SD 0.07) and mean ratings for importance of 4.40 (SD 0.71) and feasibility of 4.20 (SD 0.94); the six statements reflect the need for community capacity building and open conversations around mental health.

### Go-Zone map analysis

**Figure 4:**
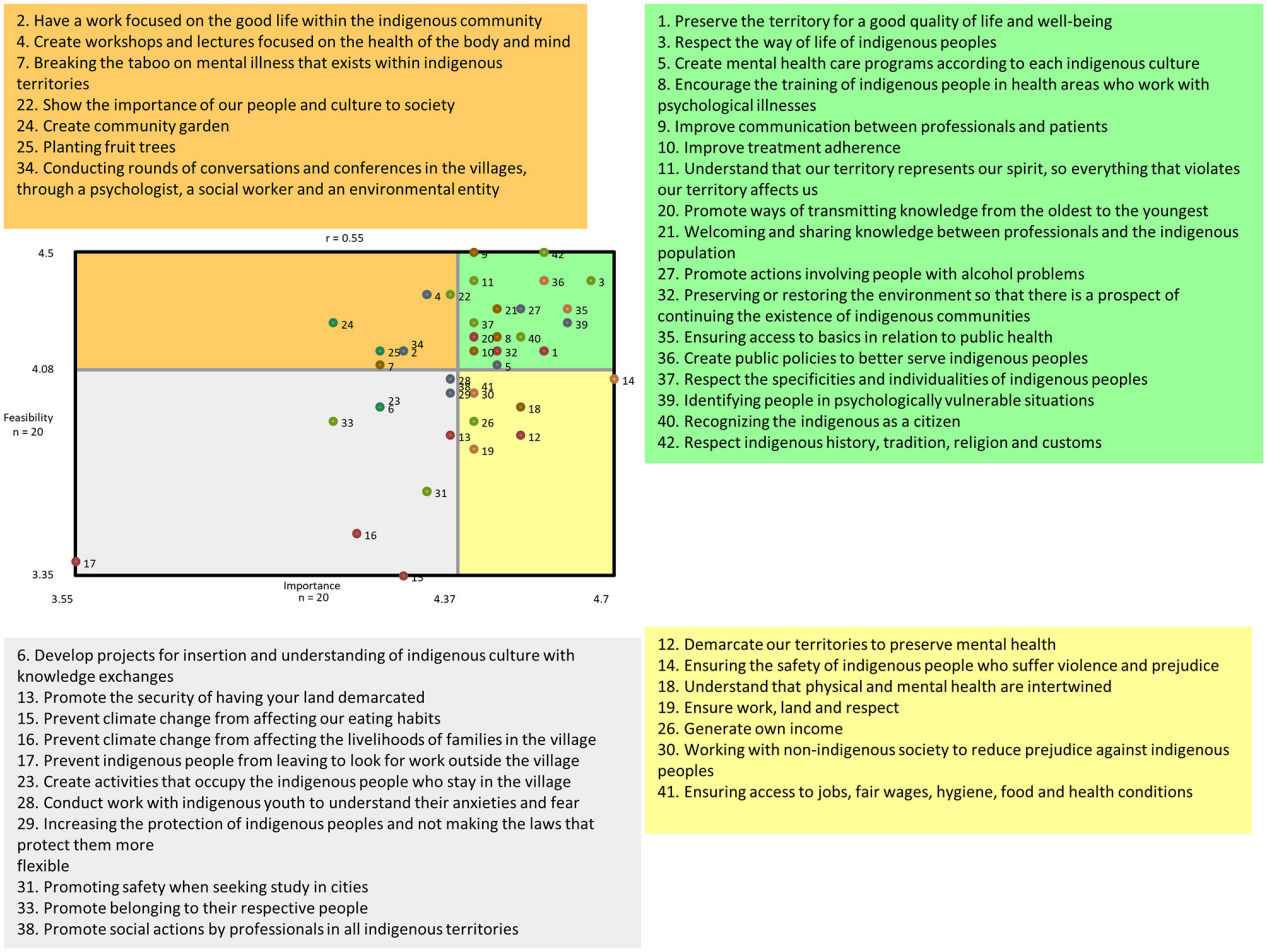
Go-Zone map illustrating the mean ratings for importance and feasibility for each statement. The colors of each point represent which cluster the statements belong to.

Figure 4 presents the four-quadrant “Go-Zone” map, which shows the participant's rating of the importance and feasibility of each statement. The x-axis represents importance, and the y-axis represents the feasibility of each statement. The upper right quadrant includes statements with above-average ratings for both importance and feasibility. These statements included those that encapsulated holistic approaches to improving Indigenous mental health, involving Indigenous communities in decision processes for equitable mental health interventions to promote a harmonious relationship between humans and nature and to continue advocating for the rights of Indigenous people across multiple sectors.

## Discussion

### Principal findings

Indigenous university students participated in concept mapping exercises to provide structured evidence about actionable priorities to improve Indigenous mental health associated with climate change. The participatory method allowed stakeholders to state and organize their thoughts, which is critical for developing culturally relevant interventions, fostering system-level change, and addressing barriers and facilitators for implementation activities. The results showed that capacity building and partnership between Indigenous and non-Indigenous communities at the systems and individual levels are critical in underpinning sustainable system-level change.

There is a growing body of research showcasing the importance of traditional Indigenous knowledge in addressing the influences of climate change on mental health. Climate change consequences often present barriers to fully observing and maintaining traditional practices for Indigenous communities worldwide (20). The relationships between Indigenous people, place, livelihoods, and culture are being altered, negatively impacting Indigenous mental health (25, 26).

Most of the evidence published on climate change comes from high-income countries, presenting an advanced discussion; some developed governments have shared recognition of the importance of Indigenous worldviews in addressing the causes and consequences of climate change (27). LMICs face multiple barriers in advancing the conversations and actions toward safeguarding Indigenous mental health as economic factors are often incentivized (28). For example, in Brazil, forests such as the Amazon are often sacrificed at the hands of the government and high-stake investors to meet agricultural demands; issues of illegal miners polluting rivers and poisoning Indigenous communities with mercury are drivers of adverse mental health experiences related to climate change (25, 29, 30).

### Impacts of cultural continuity

The impacts of climate change influence migration from traditional territories to the outskirts of cities, where many live below poverty and face significant barriers to accessing local support for health, education, housing, and social benefits. For example, the last Brazilian Institute of Geography and Statistics (IBGE) census showed that 49% of Brazilian Indigenous people live in urban centers outside demarcated Indigenous lands (4). This notable lack of basic interventions plays a significant role in the adverse health outcomes experienced by Indigenous people compared to non-Indigenous peoples (6, 9).

As ecosystems weaken in the face of climate change, Indigenous communities are adapting to pressures. These adaptations often result in the inability to fully observe traditional practices central to their cultural identity, disrupting cultural continuity, a recognized determinant of health for Indigenous people worldwide (26, 27). A revered body of research in Canada has shared that maintaining cultural practices is central to positive mental health (28–30). For example, different social roles are emerging for Indigenous women as they explain how they stray from feeding their families traditional food and do not take part in cultural activities, such as cleaning and sewing animal skins (31). In the Inuit community of Nunatsiavut, men reported a lack of confidence and nervousness about climate-related changes, whereas women reported a loss of motivation and disappointment from travel limitations (32). Young men reported that being unable to take part in hunting opportunities deprived them of the pride and sense of self-worth associated with their cultural identity, such as establishing a relationship between Indigenous cultural continuity, health, and wellbeing (33).

Consequences of food scarcity related to disruptions in marine and terrestrial ecosystems are also disruptive to cultural continuity (26–30). The inability to take part in traditional methods of hunting and gathering has influenced the migration of Indigenous to urban cities where they are more likely to encounter non-traditional foods. Five studies have reported that community members feel anxiety because of disconnection from their cultural identities and the inability to indulge in traditional foods and practices (31–35). Our co-developed conceptual models align with the literature, which all share that mental health benefits can be realized through maintaining traditional practices and a strong connection to cultural identity (32, 36).

In some developed countries, Indigenous communities find new places to hunt, build cabins, and reinforce cultural activities. They are using technology and improving infrastructure to protect themselves against extreme weather, combining traditional and Western knowledge for survival (36–38).

As reported by the stakeholders in this study, the Brazilian government should offer greater support for those migrating from traditional lands to urban centers; the conceptual framework suggests that providing financial support, jobs, access to health, and land are critical steps for improving the mental health of Indigenous people from the causes and consequences of climate change. High-income countries with Indigenous communities are taking steps necessary toward implementing Indigenous methodologies and overall place greater efforts on including Indigenous people in decisions that directly impact their communities (39).

### Capacity building

The CM highlighted the stakeholders' interest in collaboratively building public policies that directly impact mental health services. As evidence suggests, funding bodies support Western medicine models that often fail to consider cultural and historical determinants of health (40, 41). Mental health interventions are typically short-lived due to research and funding timeframes; thus, permanent programs and follow-ups with the service users are infrequently conducted (42). Through improving meaningful and active involvement from community members in health services planning and implementation, policymakers, practitioners, and community initiatives are encouraged to move toward inclusive decision-making processes and focus on the capacity building of Indigenous communities (43). Drawing from global Indigenous communities, such as in Canada, initiatives that Indigenous service users lead prove to be successful and positively impact community health and wellbeing (44, 45).

### Strengths and limitations

The study may have benefitted from a more robust sample size. While there was 100% participant retention throughout each stage of the concept mapping activities, the *n* = 20 sample size-imposed difficulties in generalizing study results.

The purpose of the participatory research was to engage young Indigenous university students to produce a visual conceptualization of ideas and their relationships regarding the mental health of Indigenous people living in their territory in association with climate change. The study shared new leanings about the actionable priorities individuals and institutions can implement to progress toward the protection of Indigenous mental health amidst climate change. The methodology championed the young Indigenous voice, which is necessary for charting the path to sustainable development of interventions for system-wide change.

The current literature base lacks consistent assessment by the Intergovernmental Panel on Climate Change (IPCC), which leads to a generic and heterogeneous literature base. Additionally, the lack of author expertise in this topic influences the saturation and overall quality of the knowledge base. Future studies should continue engaging the Indigenous people's voices and enhance the capacity of the research base.

### Future directions

We encourage future research to continue investing in the capabilities of young people to drive the development of sustainable programs in addressing mental health demands in association with climate change.

Through policies that promote respect for Indigenous social and environmental equity, equitable healthcare access, and address systemic issues that lead to disparities, Brazil and other countries alike can further align themselves with international mandates of the United Nations Declaration on the Rights of Indigenous (UNDRIP) and WHO Policy on Ethnicity and Health to restore, protect, and promote the health and wellbeing of the environment and Indigenous peoples.

## Conclusion

The mental health impacts of climate change are widely felt among Indigenous populations worldwide as their relationship to the earth is the source of spirituality, identity, and health. This CM allowed us to engage the voice of the Indigenous stakeholders through a participatory approach to identify actionable priorities to improve mental health in the face of climate change. These results show that family support, respect and understanding, improvement actions, policy actions, health, and strengthening professional skills are critical factors for sustainable interventions. The results visually reflect community knowledge and feasible priorities that can leverage a conceptual framework in policy action. The study design, data analysis, and results have provided co-developed and culturally adaptive ideas for developing and implementing equitable mental health interventions associated with climate change.

## Data availability statement

The raw data supporting the conclusions of this article will be made available by the authors, without undue reservation.

## Ethics statement

Ethical approval for this study was obtained from the National Research Ethics Commission (CONEP), Protocol CAAE 36372820.0.0000.8027 from May 25th, 2021. The study was conducted in accordance with the local legislation and institutional requirements. The participants provided their written informed consent to participate in this study.

## Author contributions

AG, ID, PJ, SH, and XZ: conceptualization, writing—review and editing, methodology, formal analysis, and writing—original draft preparation. JS, LB, MR, AV, and LR: investigation, data curation, supervision, and project administration. All authors have read and agreed to the published version of the manuscript and contributed to the article and approved the submitted version.
